# Neural correlates of motor-cognitive dual-tasking in young and old adults

**DOI:** 10.1371/journal.pone.0189025

**Published:** 2017-12-08

**Authors:** Selma Papegaaij, Tibor Hortobágyi, Ben Godde, Wim A. Kaan, Peter Erhard, Claudia Voelcker-Rehage

**Affiliations:** 1 Center for Human Movement Sciences, Groningen University of Groningen, University Medical Center Groningen, Groningen, the Netherlands; 2 Jacobs Center on Lifelong Learning and Institutional Development, Jacobs University Bremen, Bremen, Germany; 3 Brain Research Institute, University of Bremen, Bremen, Germany; Yale University, UNITED STATES

## Abstract

When two tasks are performed simultaneously, performance often declines in one or both tasks. These so-called dual-task costs are more pronounced in old than in young adults. One proposed neurological mechanism of the dual-task costs is that old compared with young adults tend to execute single-tasks with higher brain activation. In the brain regions that are needed for both tasks, the reduced residual capacity may interfere with performance of the dual-task. This competition for shared brain regions has been called structural interference. The purpose of the study was to determine whether structural interference indeed plays a role in the age-related decrease in dual-task performance. Functional magnetic resonance imaging (fMRI) was used to investigate 23 young adults (20–29 years) and 32 old adults (66–89 years) performing a calculation (serial subtraction by seven) and balance-simulation (plantar flexion force control) task separately or simultaneously. Behavioral performance decreased during the dual-task compared with the single-tasks in both age groups, with greater dual-task costs in old compared with young adults. Brain activation was significantly higher in old than young adults during all conditions. Region of interest analyses were performed on brain regions that were active in both tasks. Structural interference was apparent in the right insula, as quantified by an age-related reduction in upregulation of brain activity from single- to dual-task. However, the magnitude of upregulation did not correlate with dual-task costs. Therefore, we conclude that the greater dual-task costs in old adults were probably not due to increased structural interference.

## Introduction

Although postural control of standing is highly automated, combining standing with a cognitive task can interfere with the execution of one or both tasks [1]–[4]. Such decline in performance from single- to dual-task is called dual-task cost (DTC). Compared to young adults, old adults exhibit higher DTC in a broad array of motor and cognitive tasks. [5]–[10]. These studies propose that old adults require more attentional or processing resources for single-task performance, therefore increasing interference during dual-tasking [8]–[10]. However, the neural correlates underlying the age-related changes in dual-tasking remain unclear. An understanding of these neural mechanisms would not only help to clarify the basic concept of DTC but also strengthen the conceptual basis of interventions incorporating dual-tasking, a common practice in the promotion of old adults’ health [11]–[14]. By knowing what causes DTC in old age, interventions can be designed to target the underlying problem, for example by shifting the focus to single- or dual-task training, changing the specific tasks trained, or training particular neural regions involved.

DTC seems to arise from a competition for limited brain resources perhaps due to a limited amount of cortical tissue that can be concurrently activated [15]. This idea was supported by the finding that the total amount of brain activation during dual-tasking (number of voxels active) was less than the sum of the single-tasks even though the single-tasks evoked only non-overlapping brain areas [15], [16]. Although a limited amount of brain activation might explain a part of the DTC, the costs increase when the single-tasks require similar brain regions [17]–[20]. For example, visuospatial compared with non-spatial tasks affected old adults’ gait more severely, probably because visuospatial working memory is also involved in gait control [18]. In addition, DTC correlated with similarity in brain activation between single-tasks measured with functional magnetic resonance imaging (fMRI) [21]. The competition for shared brain regions has been called structural interference.

There is now overwhelming evidence that old compared with young adults use expanded brain networks and increased brain activation to perform a single motor or cognitive task [22], [23]. The age-related increase in neural activation may lead to greater structural interference because of reduced residual capacity in shared brain regions, causing dual-task decrements [22], [24], [25]. This hypothesis was tested in an fMRI study using an arithmetic (i.e., serial addition by three) and a visuomotor task (i.e., circle drawing), by looking at the increase (upregulation) in brain activation from single- to dual-task in shared brain regions [24]. Less upregulation in old compared with young adults would indicate greater structural interference. Overlapping brain activation was located in the supplementary motor area (SMA), which was then subjected to a region of interest analysis. SMA activation was higher in old vs. young adults during the visuomotor task and similar during the arithmetic task. However, both young and old individuals were able to upregulate their brain activation from single- to dual-task performance. Therefore, there was little or no evidence for increased structural interference. However, age also did not affect DTC, suggesting that the arithmetic task was perhaps not challenging enough, which made it difficult to draw any conclusions. Our goal was therefore to determine whether increased structural interference underlies dual-task decrements in old adults using more challenging tasks.

Another often-debated aspect of dual-tasking is whether it requires additional brain activation that cannot be explained by the single-tasks. Some studies did find evidence for such dual-task specific activation [26]–[29], whereas others did not [17], [30], [31]. To the best of our knowledge, only one study investigated dual-task specific activation in young and old adults, finding no dual-task specific activation in either of the age groups [24]. Therefore, our second goal was to determine whether there is dual-task specific activation in a challenging motor-cognitive dual-task, and if so, whether there are age-related changes in dual-task specific activation.

Dual-tasking can comprise a diverse repertoire of composite tasks, including working memory, arithmetic, visuomotor, reaction time, and balance tasks. Gait speed in combination with the ability to maintain one’s balance are hallmarks of mobility in old age [32]–[34], and dual-task decrements in motor-cognitive balancing tasks are associated with an increased fall risk [35]–[38]. Therefore, we simulated active balance control in the MRI scanner using a plantar flexion force control task in the context of maintaining one’s standing balance, a task that activates brain regions observed also in active and imaginary standing [39]–[43], and correlates with postural sway during quiet standing [44], [45]. We paired simulated balancing with a challenging cognitive task, serial subtraction by seven, to increase dual-task challenge and induce a more robust structural interference than reported previously [24]. The arithmetic task was used to allow comparison with the previous study [24], because of its continuous nature and because it does not require a visual input. Although balance-cognitive dual-tasks have been studied extensively at behavioral level in young and old adults [46], this study is the first to examine the neural correlates of balance-cognitive dual-task interference using fMRI.

We hypothesized that old versus young adults would exhibit: (1) lower performance and higher brain activation during single-tasks; (2) reduced upregulation in brain activation from single- to dual-task in shared brain regions, due to the already high activation during single-tasks, accompanied by greater DTC at a behavioral level, and (3) no dual-task specific activation in either age group [24]. A negative correlation between the magnitude of upregulation and dual-task costs would further support the hypothesis that an increase in structural interference underlies the age-related decline in dual-task performance. Based on previous imaging studies on balance control [39]–[42], [47] or mental calculation [24], [48]–[51], potential regions for structural interference were the SMA, insula, thalamus, precuneus, prefrontal gyrus, middle/inferior frontal gyri, and inferior parietal lobule.

## Materials and methods

### Participants

Twenty-six young female adults (mean ± SD age 23.6 ± 2.6 years, range 20–29) and 42 old female adults (mean ± SD age 73.9 ± 5.1 years, range 66–89) participated in the study. The participants had no history of neurological disorders, severe cardiovascular diseases, or severe orthopedic disorders, and were not under psychoactive medication. For practical reasons, old adults were recruited from an all-female database created in a previous study at our center [52]. To allow comparison between young and old adults, only female young adults were recruited via advertisements at the Jacobs University and Bremen University, Germany. Before the experiment, all subjects signed an informed consent document. The Medical Ethics Committee of the German Association of Psychology specifically approved this study. Three young and ten old adults were excluded from the analyses, because of technical issues (n = 4), task-related movement in the scanner (n = 1), visual problems (n = 1) or calculation below chance level (n = 7). Movement in the scanner was assessed by calculating the root mean square head position change (< 1.5mm) and visual inspection of the realignment plots (movements related to the tasks). Visual problems contained self-reported difficulty with reading the numbers in the scanner (despite the provided glasses). Chance level was defined as having one third of all calculation trials correct, including single and dual-task conditions. Table 1 shows the characteristics of the included and excluded participants. We assessed the old adults’ short-term memory, visuospatial abilities, executive functions, attention, concentration, working memory, language, and orientation, using the Montreal Cognitive Assessment (MoCa). Compared with MoCa normative data our participants’ cognitive ability was just above average [53]. Four old adults had a MoCa score between 20 and 24, which would indicate mild cognitive impairment when using the 24/30 cut-off score [54], [55], though not when using the 20/30 cut-off score [56]. Because they performed within the normal range (as defined by the outlier labeling rule) on dual-task costs and structural interference, we included them in the analyses.

**Table 1:** Subject characteristics. Values are mean ± SD, with the range between brackets. MoCa: montreal cognitive asessment (max. score of 30).

	Young		Old	
	Included (n = 23)	Excluded (n = 3)	Included (n = 32)	Excluded (n = 10)
Age (years)	24 ± 3 (20–29)	23 ± 3 (21–26)	74 ± 5 (66–89)	75 ± 4 (69–81)
MoCa score	-	-	26 ± 3 (21–30)	26 ± 2 (23–30)
One leg stance time (s)	19.8 ± 1.0 (15.4–20.0)	18.3 ± 2.9 (15.0–20.0)	6.0 ± 2.7 (2.1–11.6)	7.8 ± 5.2 (3.9–20.0)
Years of education	17 ± 2 (14–21)	17 ± 1 (16–18)	14 ± 3 (8–19)	13 ± 2 (10–15)

### Experimental setup

**Fig 1:**
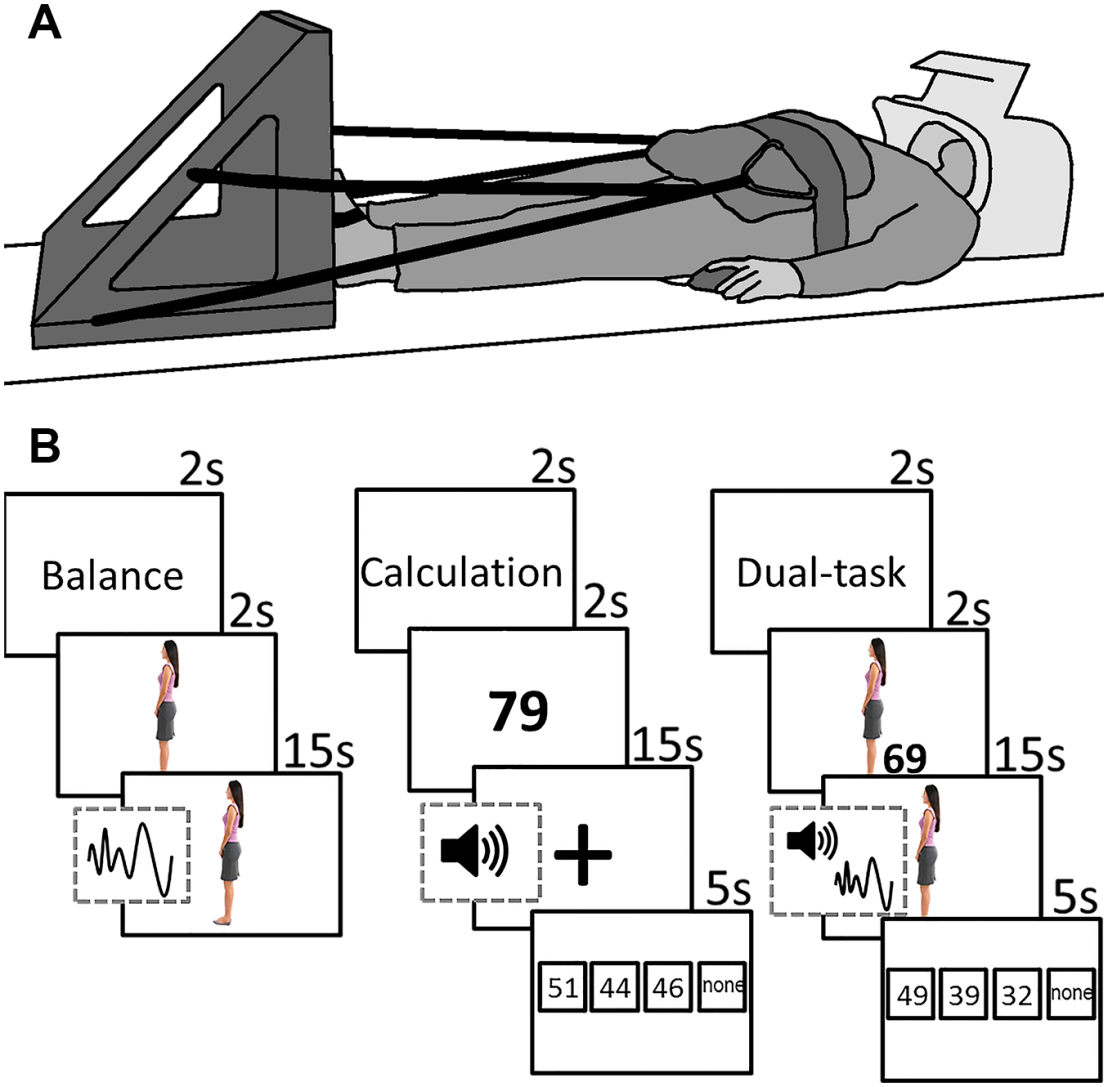
**A:** Illustration of experimental setup in the scanner. Participant lies supine with her feet against the force platform. **B:** Schematic of the stimuli during balance, calculation and dual-task conditions. The sine wave signifies the addition of the disturbance signal, causing the avatar to sway forward and backward. The speaker signifies the addition of beeps, which indicated the time points at which the participant had to subtract.

Participants lay supine in the MRI scanner with their feet against a custom-made force platform attached to the MRI bed (Fig 1A). The position of the force platform was adjusted to subject height via a sliding mechanism. The force platform consisted of a sturdy plastic board connected to a load cell (Vishay Tadea-Huntleigh Model 1402, Oisterwijk, the Netherlands) positioned between the two feet at the height of the first metatarsophalangeal joints. The feet were affixed to the force platform by Velcro straps. To simulate weight-bearing and avoid excessive head movement, participants were pulled towards the force platform using thick elastic ropes attached to a hip belt (C.P. Sports Ultraleichtgürtel, Germany). The amount of axial load was set to 70–80 N. A four-button device was placed underneath the right hand for the calculation task.

We created a virtual instrument using National Instruments LabVIEW 2013 (13.0.1f2) to acquire the force data with a sample frequency of 100 Hz, and display the tasks to the participants. The tasks were projected onto a white screen placed at the head of the scanner. Participants could see the screen via a mirror attached to the head coil.

### Tasks

During the balance task, an avatar in the shape of a woman was displayed on the screen. The avatar swayed forward and backward. Participants were instructed to try to keep the avatar in the upright position by increasing or decreasing the level of plantar flexion force measured by the load cell. As in normal standing, increasing the plantar flexion force led to a backward sway, and vice versa. The avatar was upright at a force of 75 N and swayed with 1.8°/N. These numbers were chosen based on pilot data to minimize head movement while still requiring active force control. Force data were filtered online with a 10-point moving average filter. At the start of every balance condition, participants were given two seconds to bring the avatar in the upright position. After these two seconds, a disturbance signal was added, causing the avatar to sway forward and backward. In order to keep the avatar upright, participants had to counteract these disturbances. The disturbance signal was made by combining fifteen sinusoidal signals with random phases and with frequency characteristics based on an average frequency spectrum of Center of Pressure movement during upright standing (0.025–1 Hz), measured in ten young and ten old adults. The maximum amplitude of the disturbance was ±30°.

The calculation task consisted of serial subtractions with increments of seven. At the start, a number between 50 and 100 was projected on the screen for two seconds. Every participant received the same set of start numbers (calculation only: 79, 66, 96, 62, 54, 60, 73, 58, 97, 65, 51, 82; dual-task: 58, 90, 69, 61, 87, 76, 89, 86, 95, 63, 72, 55). Then, a plus sign was displayed on the screen and every 3 to 4 seconds a beep was generated through an MRI compatible headphone (MR confon Optime 1, Magdeburg, Germany), with a total of four beeps per trial. Participants were instructed to subtract seven at every beep. At the end, four answer possibilities were given, always including the correct answer, two erroneous answers, and the option that none of the other answers is correct. Participants indicated which answer they thought was correct by pressing the corresponding button of the four-button device.

During the dual-task condition, subjects performed the balance and calculation tasks simultaneously. They were instructed to do both tasks as well as possible. All participants received a short practice session outside of the MRI scanner and again in the scanner, to make sure that the instructions were understood. During the first practice session participants were asked to count out loud, to verify that they were able to perform the calculations and minimize the chance of guessing during the experiment.

After the experiment, participants were asked for their calculation strategy during single- and dual-tasking. All participants subtracted seven after each beep, except for one young and one old adult, who counted the beeps and subtracted the total number at the end. The calculation strategy was thus similar between age groups and conditions.

### Experimental design

An fMRI block-design was used to alternate between the three conditions: balance, calculation, and dual-task. Every participant performed twelve blocks, each block including one trial of each condition (thus three trials), with the order of the conditions balanced across blocks and randomized between subjects. At the end of every block a 15-second rest period was given in which the participants fixated their gaze on a plus sign. The number and duration of blocks was designed in accordance with general guidelines to ensure sufficient power to detect the effects of interest [57]. Fig 1B illustrates the time frame of the visual stimuli per condition.

### Behavioral data analysis

Performance on the balance task was quantified as the root-mean-square error (RMSE) of the difference in angle between the avatar and its vertical position, averaged across blocks. Performance on the calculation task was quantified as the percentage of correct answers (%correct). DTC was calculated for the balance and calculation tasks using the following formula [24]:

$$DTC\;=\;\frac{Performance(single-task)\;-\;Performance(dual-task)}{Performance(single-task)}\times 100$$

High DTC scores denote a great decline in performance from single- to dual-task. Mean DTC was calculated by averaging the DTC for the balance and calculation tasks [58].

### Statistical analysis of behavioral data

All behavioral variables (RMSE, %correct, balance DTC, calculation DTC, mean DTC) were checked for outliers using the outlier labeling rule [59] with a multiplier of 2.2 [60]. Mean DTC in two young participants were identified as outliers and these data points were excluded from further analyses. An age (young, old) by condition (single-task, dual-task) mixed ANOVA was performed on RMSE and %correct. An independent t-test was used to test differences in mean DTC between young and old adults. The alpha level was set at 0.05.

### Validity of simulated balance

In a separate experiment and subject pool, we determined the validity of balancing the avatar in the supine position in relation to the fluctuation of the center of pressure while standing. Young (n = 12, age 21.3 ±1.60) and healthy old adults (n = 7, age 69.8 ±4.38) stood on a force platform as still as possible for two minutes and looked at a 30 by 30 cm white cross, projected on a 3 by 2 m white screen 1.5 m away at eye level. Subjects also performed the simulated balance task for two minutes. As in the MRI machine they were tied with elastic ropes to a force platform (Fig 1) and kept the avatar vertical by pressing on the platform while lying on an examination table. The data revealed a correlation of r = 0.64 (p = 0.003) between the root mean square error in the simulated balance task and the center of pressure velocity measured during standing. These data suggest a reasonable at least contextual validity of the simulated balance task in the supine position in relation to real balancing in a vertical standing position.

### fMRI data acquisition

Brain imaging was performed on a 3-T SIEMENS Magnetom Skyra System (Siemens, Erlangen, Germany) with a 20 channel head/neck coil. For functional scans, A T2*-weighted multiband gradient echo-planar imaging (EPI) sequence was used (TR = 700 ms, TE = 30 ms, flip angle = 55°, 48 axial slices, slice thickness = 3 mm, no gap, in-plane resolution 3x3 mm) (Feinberg et al., 2010). After the functional scanning session, a high resolution magnetization prepared rapid acquisition gradient echo (MPRAGE) T1-weighted sequence (TR = 2100 ms, TE = 4.6 ms, TI = 900 ms, flip angle = 8°, 192 contiguous slices, voxel resolution 1 mm³, FOV = 256x256x192 mm, iPAT factor of 2) was obtained in sagittal orientation. These anatomical scans were used to co-register the functional runs.

### fMRI data analyses

#### Preprocessing

All fMRI data analyses were performed using the statistical parametric mapping software SPM 12 (Welcome Department of Cognitive Neurology, London, UK), implemented in Matlab R2014b (Mathworks, Natick, MA). For each subject, we corrected for inter-scan movement using the realign and unwarp option with the first scan as a reference. The anatomical scan was segmented using the SPM tissue probability maps. All functional scans were co-registered to the anatomical scan and normalized to the Montreal Neurological Institute (MNI) template brain via the forward deformations revealed by the segmentation. The normalized images were smoothed using an 8-mm FWHM Gaussian kernel.

#### First level fMRI analysis

All analyses were performed using the General Linear Model. Each condition was modeled with a boxcar function convolved with a canonical hemodynamic response function (HRF). Also the pre- and post-condition episodes (condition announcement, start balance, number display, button response) were entered separately into the model. The motion parameters obtained from the realignment step were used as covariates. A high-pass filter (1/277 Hz) was applied to remove low-frequency signal drifts and a first-order autoregressive model was fit to the residuals to account for temporal correlations. For each subject, condition (balance, calculation, dual-task) vs. baseline contrasts were computed.

#### Group level fMRI analysis

The first-level contrasts were subjected to a random-effects analysis. A 2x3 full factorial design was used with age-group as between-subjects factor (young, old) and condition as within-subjects factor (balance, calculation, dual-task). Brain activation associated with each condition was assessed by computing the simple effects. Age effects on brain activation were assessed by computing the t-contrasts ‘young vs. old’ and ‘old vs. young’ for each condition. The statistical threshold was set at p < 0.05, family wise error (FWE) corrected at the voxel level. Brain regions were labeled using automated anatomical labeling [Tzourio-Mazoyer et al., 2002].

#### Age effects

To test whether age-related changes in activation were related to performance, we performed regression analyses with brain activation associated with balance, calculation or dual-tasking as dependent variable and single-task performance and balance, calculation, and mean DTC as covariates of interest (p < 0.001 uncorrected, extend threshold of 10 voxels). The corresponding ‘young > old’ and ‘old > young’ contrasts (p < 0.001, uncorrected) were used as inclusive masks.

#### Dual-task specific activation

To test whether dual-tasking required additional activation of brain regions that cannot be explained by the single-tasks, we calculated the contrast per age group [61]. This contrast subtracts the sum of the single-task activations from the dual-task activation. The contrast ‘dual-task > baseline’ was used as an inclusive mask (p < 0.001, uncorrected). Significant differences were recognized at p < 0.05, FWE corrected.

#### Structural interference

To find the brain regions with potential structural interference between balancing and calculation, we performed a conjunction analysis with four contrasts: ‘balance vs. baseline’ and ‘calculation vs. baseline’ for young and old adults (p < 0.05, FWE corrected). A conjunction analysis of these four contrasts revealed those brain regions where for both age groups’ activations for both single tasks overlapped, i.e., where structural interference could be expected. These brain regions were subjected to a ROI-analysis. To minimize the number of ROI-analyses, the extent threshold was set at ten voxels. We extracted the beta values from the individual peak voxels for the conjunction analysis within every ROI. An age (young, old) by condition (balance, calculation, dual-task) repeated measures ANOVA was performed on these peak voxels for every ROI to see if there were any age by condition interaction effects. In case of violation of the assumption of sphericity, a Greenhouse–Geisser correction was applied. The Bonferroni method was used to correct for multiple comparisons.

#### Differences in up-/downregulation

We performed post-hoc analyses in those ROI’s that showed a significant age by condition interaction. As we expected, old compared with young adults revealed an increase in activation in terms of increased BOLD signals, i.e., higher beta values, during single-tasks. Therefore, there was less residual capacity to upregulate the activation for the dual-task from the single tasks. Using the extracted beta values, we calculated the upregulation from single-tasks to dual-task as follows:

$$\text{Upregulation}=\frac{\text{BOLD}(\text{dual-task})-\text{BOLD}(\text{mean}\;\text{of}\;\text{both}\;\text{single-tasks})}{\text{BOLD(mean}\;\text{of}\;\text{both}\;\text{single-tasks)}}\times 100$$

Mean upregulation was calculated by averaging the upregulation per ROI. Outliers were detected according to the outlier labeling rule (cf. above) and excluded from analysis (1.4% of all data points). Independent t-tests were used to compare the upregulation between age groups. P-values were corrected for multiple comparisons. Pearson correlations were computed between upregulation and balance DTC, calculation DTC, and mean DTC.

## Results

### Behavioral data

**Fig 2:**
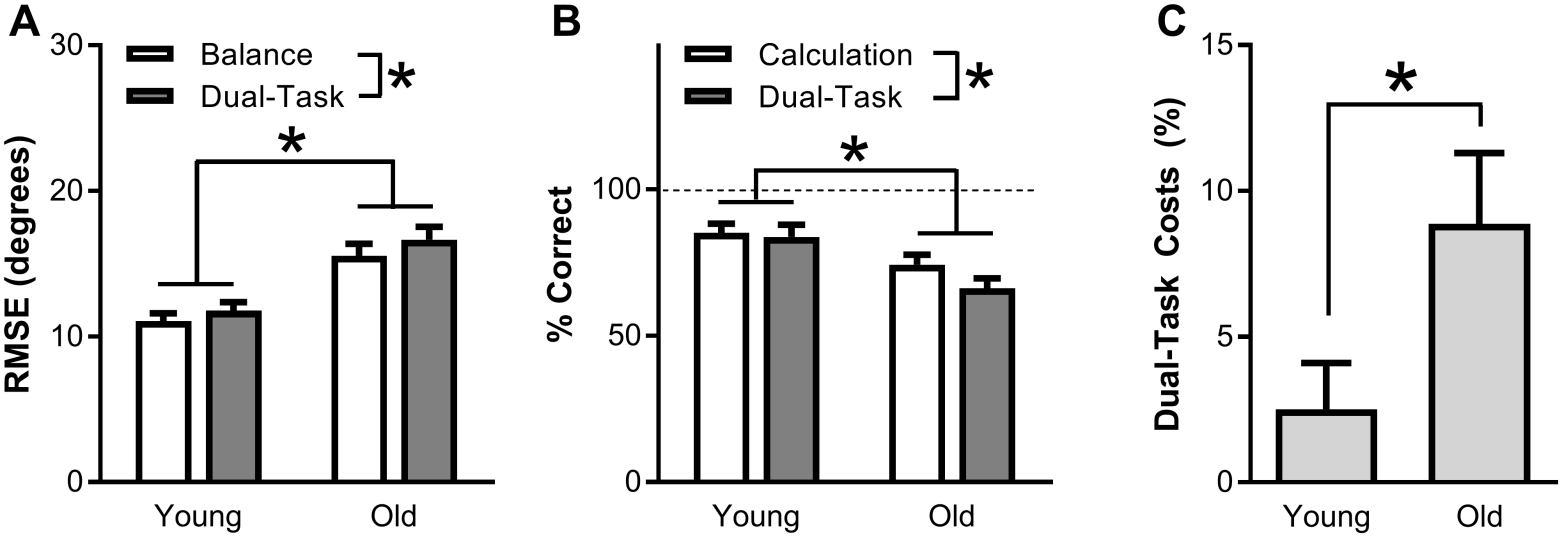
Behavioral group data for young and old adults, showing the root-mean-square error (RMSE) angle between the avatar and the vertical during balance and dual-tasking (A), percentage trials with a correct answer on the calculation task during calculation and dual-tasking (B), and performance decline from single- to dual-tasking, averaged over the balance and calculation tasks (C). Asterisks denote statistical significance (p<0.05).

Fig 2A shows the group data for the performance on the balance task during the balance-only task and balance plus calculation dual-task. Balance performance was significantly worse during dual- vs. single-tasking, as shown by a 7% increase in RMSE (F(1,53) = 9.0, p = 0.004, $\eta_{p}^{2}\;=\;0.15$). Across conditions, RMSE was 41% higher for old compared with young adults (F(1,53) = 18.7, p < 0.001, $\eta_{p}^{2}\;=\;0.26$). There was no significant age x condition interaction in RMSE (F(1,53) = 0.5, p = 0.504, $\eta_{p}^{2}\;=\;0.01$).

Fig 2B shows the data for the percent of correct calculation trials during single- and dual-tasking. Overall, similar to balancing, the calculation performance decreased by 7% between dual- and single-tasking (F(1,53) = 5.5, p = 0.022, $\eta_{p}^{2}\;=\;0.10$) and was 17% lower in old vs. young adults (F(1,53) = 9.3, p = 0.003, $\eta_{p}^{2}\;=\;0.15$). Again, there was no significant age x condition interaction (F(1,53) = 2.6, p = 0.111, $\eta_{p}^{2}\;=\;0.05$).

Mean DTC was calculated by averaging the performance decline from single- to dual-tasking for the balance and calculation task. Mean DTC was significantly higher in old (9%; 9% for both balance and calculation) than in young adults (2%; 6% for balance, -1% for calculation) (t(49) = -2.1, p = 0.037, r^2^ = 0.10, Fig 2C).

### Condition effect on neuroimaging data

**Fig 3:**
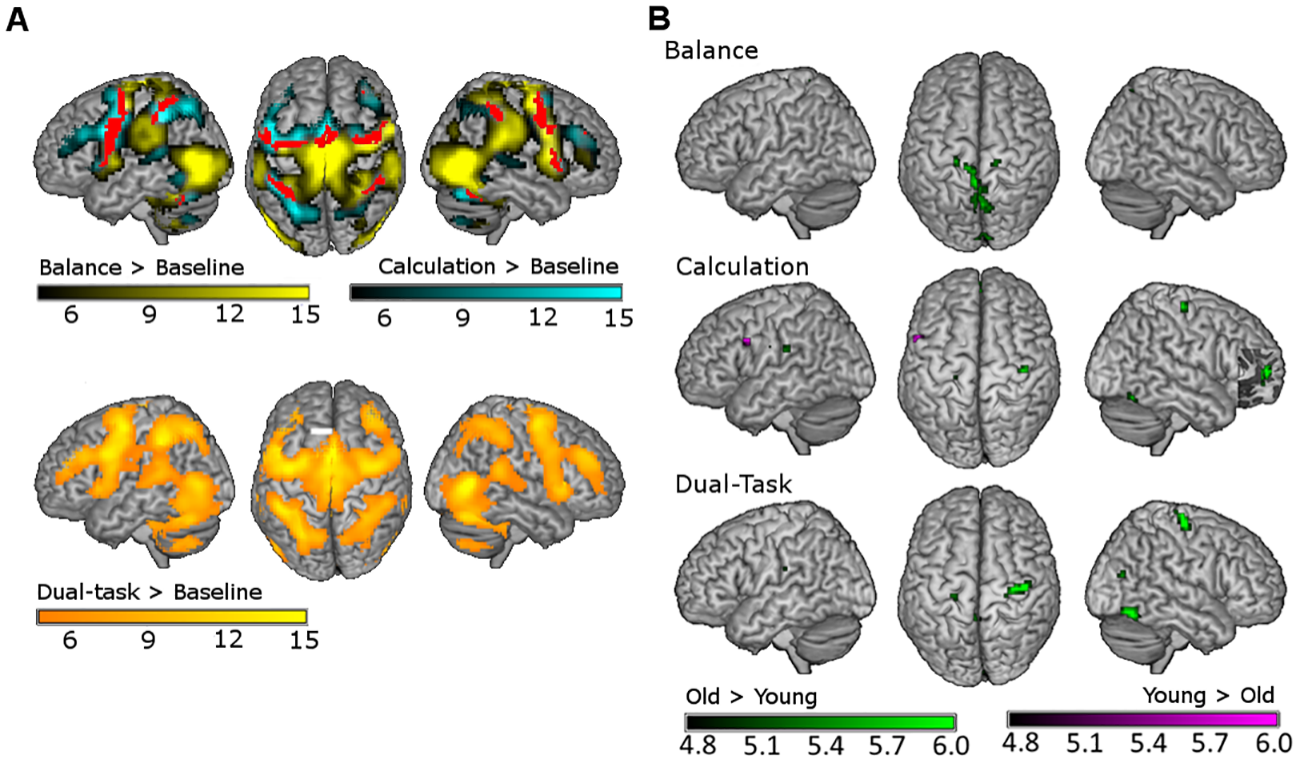
**A:** Mean brain activation patterns during balance and calculation (upper panel), and dual-tasking (lower panel) as compared with baseline. Significantly activated voxels during balance, calculation and dual-tasking, i.e. the main task effects, are shown in yellow, cyan and orange colors, respectively. Voxels shown in red in the upper panel are those revealed by conjunction analysis (balance AND calculation) and therefore represent areas not specific for a single-task. **B:** Age-related differences during balance, calculation, and dual-tasking. Voxels with higher activation in old compared with young adults are shown in green, whereas voxels with higher activation in young compared with old adults are shown in purple. Statistical significance was set at p < 0.05, FWE corrected for multiple comparisons.

Table 2 lists the local maxima of activated clusters during balance, calculation and dual-task conditions. Fig 3A shows activation patterns as compared to baseline. Deactivation patterns are described in more detail in the supplementary material. Main areas that were activated during balance were the bilateral MT/V5 areas (on the border between middle temporal gyrus and middle occipital gyrus), motor areas (SMA, bilateral precentral gyri), somatosensory areas (postcentral gyri, paracentral lobules, supramarginal gyri), frontal areas (middle and inferior frontal gyri), subcortical areas (cerebellum, caudate nucleus, putamen, thalamus), and the insular cortices. Deactivations were evident mainly in occipital areas (left cuneus, bilateral calcarine sulci, and left lingual gyrus).

**Table 2:** MNI coordinates and t-values of the local maxima with significant activation during balance, calculation and dual-tasking (p < 0.05; FWE corrected for multiple comparisons). Voxel size is 3x3x3 mm.

AAL location	Side	Dual-task				Balance				Calculation			
		MNI coordinates			t-value	MNI coordinates			t-value	MNI coordinates			t-value
		x	y	z		x	y	z		x	y	z	
Superior frontal gyrus (orbital part)	L	-21	51	-9	5.0								
Superior frontal gyrus	R									27	3	54	11.3
Middle frontal gyrus	L	-24	0	54	13.9	-36	33	33	5.4	-24	3	54	14.4
	R	39	-3	60	13.9	39	-6	57	17.0				
Inferior frontal gyrus (pars opercularis)	L	-57	6	18	11.3	-54	6	18	11.2				
	R	51	12	12	9.3	54	12	12	15.6				
Inferior frontal gyrus (pars triangularis)	L									-42	30	27	11.9
	R									42	33	30	10.9
Insula	L	-30	24	3	11.7	-45	3	6	12.6	-30	24	3	12.9
	R	33	21	6	13.4	45	12	0	12.4	33	24	6	13.2
Midcingulate area	L					-12	-24	45	9.9				
	R					12	-24	45	9.2				
Precentral gyrus	L	-39	-6	54	15.4	-39	-9	54	15.0	-45	6	33	16.7
	R	42	0	45	11.0	57	6	30	15.7	57	3	21	6.0
SMA	L	-3	-6	66	13.8	-3	-12	66	20.0	-3	9	54	17.6
	R	3	12	51	17.2								
Paracentral lobule	L	0	-21	69	12.7	0	-21	69	21.8				
	R					9	-39	66	12.7				
Postcentral gyrus	L	-48	-18	33	6.2	-33	-39	57	11.8				
	R	30	-36	54	8.5	30	-36	54	14.2				
Superior parietal lobule	L	-27	-63	51	13.7					-27	-63	51	15.6
	R	30	-51	57	10.5	30	-48	57	13.0				
Inferior parietal lobule	L	-42	-39	42	15.6					-42	-39	42	17.4
	R	39	-39	45	10.2					48	-36	48	11.0
Rolandic operculum	R	42	-27	21	6.5								
Precuneus	L									-12	-69	57	10.9
	R	12	-69	54	8.1					12	-66	54	8.4
Supramarginal gyrus	L	-48	-36	24	8.9	-48	-33	24	12.9				
	R					57	-30	27	15.0				
Superior temporal gyrus	L	-60	-39	15	10.2					-60	-39	12	9.8
	R	60	-39	18	10.7					57	-36	12	7.2
Middle temporal gyrus	L									-54	-45	-12	7.5
	R	45	-66	6	17.4	48	-69	3	24.6				
Inferior temporal gyrus	L	-51	-60	-9	10.1					-51	-57	-9	9.6
	R					42	-54	-15	15.6	57	-48	-12	7.3
Superior occipital gyrus	L					-24	-75	33	5.9				
Middle occipital gyrus	L	-42	-72	6	12.8	-45	-72	3	20.5				
	R	30	-72	30	7.0	30	-72	30	10.1				
Thalamus	L					-12	-15	9	6.2				
	R					12	-15	9	8.1				
Brain Stem						6	-27	-36	5.4				
Caudate nucleus	L	-12	-6	15	8.3	-12	-6	15	6.3	-15	-3	15	7.1
	R	15	-6	18	8.9					15	-6	18	6.6
Putamen	L	-18	6	9	8.0	-27	-3	9	10.8	-21	9	6	6.9
	R	27	-9	12	5.6	30	0	9	9.1				
Globus pallidum	R	15	0	0	6.3								
Cerebellum (lobule 3)	R	15	-36	-24	6.6	15	-36	-24	9.2				
Cerebellar vermis (lobule 4–5)		0	-48	-6	9.8	0	-48	-6	10.0	0	-54	-15	7.0
Cerebellum (lobule 4–5)	L	-15	-36	-24	9.0	-15	-36	-24	11.4				
	R	30	-33	-33	8.4	30	-36	-30	8.5				
Cerebellar vermis (lobule 6)		6	-75	-18	12.4	0	-72	-18	7.3				
Cerebellum (lobule 6)	L	-33	-57	-27	12.4	-33	-51	-27	13.0	-33	-57	-30	11.7
	R	33	-69	-24	12.9	18	-69	-21	6.2	33	-69	-24	13.5
Cerebellar vermis (lobule 7)		0	-72	-33	11.2								
Cerebellum (lobule 7b)	L					-12	-72	-45	11.5				
Cerebellar vermis (lobule 8)						6	-72	-42	10.5				
Cerebellum (lobule 8)	L	-30	-63	-45	9.3	-33	-51	-51	9.9	-30	-66	-48	8.6
	R	24	-69	-48	8.1	30	-42	-48	7.7	24	-69	-48	9.3
Cerebellum (lobule 9)	L	-15	-51	-42	5.1	-15	-48	-51	9.0				
Cerebellum (lobule 10)	L	-27	-36	-42	7.7								
Cerebellar hemisphere (crus I)	L									-36	-42	-36	5.2
Cerebellar hemisphere (crus II)	L									-6	-75	-30	8.5
	R									6	-75	-33	8.1

During calculation, a large fronto-parietal network was activated, including the left SMA, bilateral precentral gyri, bilateral inferior and left superior parietal lobules, left middle frontal gyrus, right superior frontal gyrus. Also bilateral insular cortices, temporal gyri, caudate nuclei and parts of the cerebellum were active. During calculation widespread areas became deactivated, including bilateral medial and superior frontal areas, bilateral angular gyri, left cingulate gyrus, left superior and bilateral middle occipital gyri, and temporal areas.

Dual-tasking evoked an activation pattern combining those of the calculation and balance task and a deactivation pattern similar to that during calculation.

### Age effect on brain activation

Table 3 and Fig 3B display the local maxima of clusters with significantly different activations between young and old adults. During balance and dual-tasking, there were no brain regions for which the young adults showed higher activation than the old adults. However, during calculation, more activation was observed in the left precentral gyrus and SMA for young than for old adults.

**Table 3:** MNI coordinates and t-values of the local maxima with significant age-related differences in activation during balance, calculation and dual-tasking (p < 0.05; FWE corrected for multiple comparisons). Voxel size is 3x3x3 mm.

AAL location	Side	Dual-task				Balance				Calculation			
		MNI coordinates			t-value	MNI coordinates			t-value	MNI coordinates			t-value
		x	y	z		x	y	z		x	y	z	
**Young > Old**													
Supplementary motor area	L									0	12	45	4.8
Precentral gyrus	L									-54	9	33	5.8
**Old > Young**													
Medial frontal gyrus	L	0	57	6	5.1					0	57	6	6.6
Precentral gyrus	L	-24	-24	60	5.3					-45	-12	27	5.8
	R	39	-18	66	6.3	15	-27	69	5.3	39	-18	63	5.8
Paracentral lobule	L	-9	-33	78	4.8	-9	-30	72	5.6				
Postcentral gyrus	L	-48	-12	27	4.9					-39	-15	39	5.0
Precuneus	L	-3	-45	72	5.3	-3	-42	72	5.8				
	R					3	-63	63	5.6				
Angular gyrus	R									42	-54	27	4.9
Supramarginal gyrus	L	-63	-24	24	5.0					-63	-24	24	5.3
Superior temporal gyrus	L	-63	-15	3	4.9								
Middle temporal gyrus	L	-45	-60	15	5.2					-45	-60	15	5.0
Middle occipital gyrus	L	-42	-78	24	5.0								
	R	42	-72	18	5.4								
Inferior occipital gyrus	R	48	-66	-15	6.6								
Calcarine sulcus	L	3	-93	-6	5.1								
Lingual gyrus	L	-3	-87	-15	5.6								
	R	12	-93	-9	5.3								
Cuneus	L					-3	-93	15	5.5				
	R					9	-93	15	5.3				
Fusiform gyrus	R									48	-63	-18	5.6
Cerebellum (Crus I)	L									-12	-90	-21	4.9

In all three conditions, there were clusters with significantly higher activation in old as compared with young adults. During balance, old adults showed higher activation in motor (right precentral gyrus and left paracentral lobule) and sensory regions (bilateral precuneus and cuneus). During calculation, main clusters with age-related activation increases were found in the bilateral precentral gyri and the left medial frontal gyrus. These areas were also more activated in old adults during dual-tasking. Additionally, bilateral lingual gyri, right inferior and middle occipital gyrus, left calcarine sulcus, and left precuneus were more activated in old than in young adults during dual-tasking. Bar plots of the BOLD responses in the clusters with an age-related increase in activation during dual-tasking can be found in the supplementary material. Using a multiple regression model, there were no significant relationships between brain activation and behavioral performance (single-task performance and DTC) in the brain regions showing age-related changes in brain activation.

### Dual-task specific activation

Table 2 shows that most brain regions involved in dual-tasking are also involved in balance or calculation, suggesting little to no dual-task specific activation. Indeed, when contrasting the dual-task condition with the sum of the single-task conditions, there were no significant clusters in either age group.

### Structural interference

**Fig 4:**
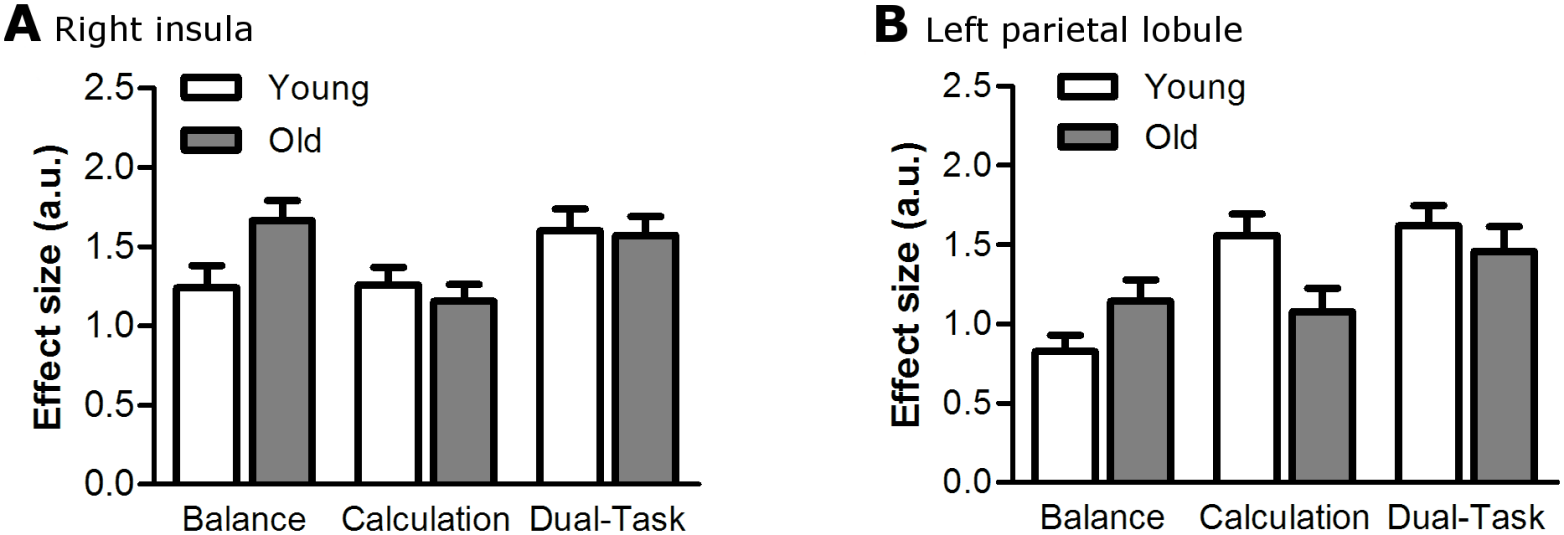
Group data for young and old adults, showing the mean ± standard error of the BOLD response (in arbitrary units) during balance, calculation, and dual-tasking, in the right insula (A) and left parietal lobule (B).

A conjunction analysis revealed eight clusters that were activated during both single-tasks in both age groups, including the SMA, right insula, bilateral premotor areas, cerebellum, and parietal lobules (Table 4). These clusters were subjected to a ROI-analysis. After multiple comparisons correction, there was a significant condition effect in all ROI’s (p < 0.024) but no significant age effect in any of the ROI’s (p > 0.104). The age by condition interaction was significant in two ROI’s: the right insula (F(2,83) = 7.0, p = 0.024, $\eta_{p}^{2}\;=\;0.12$) and the left parietal lobule (F(1,68) = 9.4, p = 0.008, $\eta_{p}^{2}\;=\;0.15$) (Fig 4).

**Table 4:** MNI coordinates and t-values of the local maxima from the conjunction of the balance and calculation contrasts. Cluster size is given for the peak voxel per cluster. Voxel size is 3x3x3 mm. P-values for the age (young, old) by condition (balance, calculation, dual-task) interactions are given, with and without Bonferonni correction.

AAL location	Side	Cluster size	MNI coordinates			t-value	Age x Condition interaction	
			x	y	z		unc. P-value	corr. P-value
Middle frontal gyrus	R	136	30	0	57	6.8	0.069	0.552
Precentral gyrus	R		39	0	42	6.5		
Inferior frontal gyrus (pars opercularis)	R		45	9	30	5.3		
Precentral gyrus	L	233	-54	6	18	7.7	0.174	1.000
Superior frontal gyrus	L		-24	-6	57	6.1		
Inferior frontal gyrus (pars opercularis)	L		-48	9	6	5.7		
Superior frontal gyrus	L		-24	-3	69	5.3		
Insula	R	61	33	18	6	6.9	0.003*	0.024*
Supplementary motor area	L	145	0	0	60	7.8	0.060	0.480
	R		3	6	54	7.7		
Superior parietal lobule	L	52	-42	-42	57	5.8	0.001*	0.008*
Inferior parietal lobule	L		-48	-33	42	5.6		
Inferior parietal lobule	R	62	48	-36	48	6.0	0.007*	0.056
Cerebellum (Lobule VI)	R	12	36	-54	-27	6.1	0.816	1.000
Cerebellum (Crus I)	L	22	-36	-54	-30	6.8	0.163	1.000

* denotes significant interaction with p < 0.05.

#### Up-/downregulation

**Fig 5:**
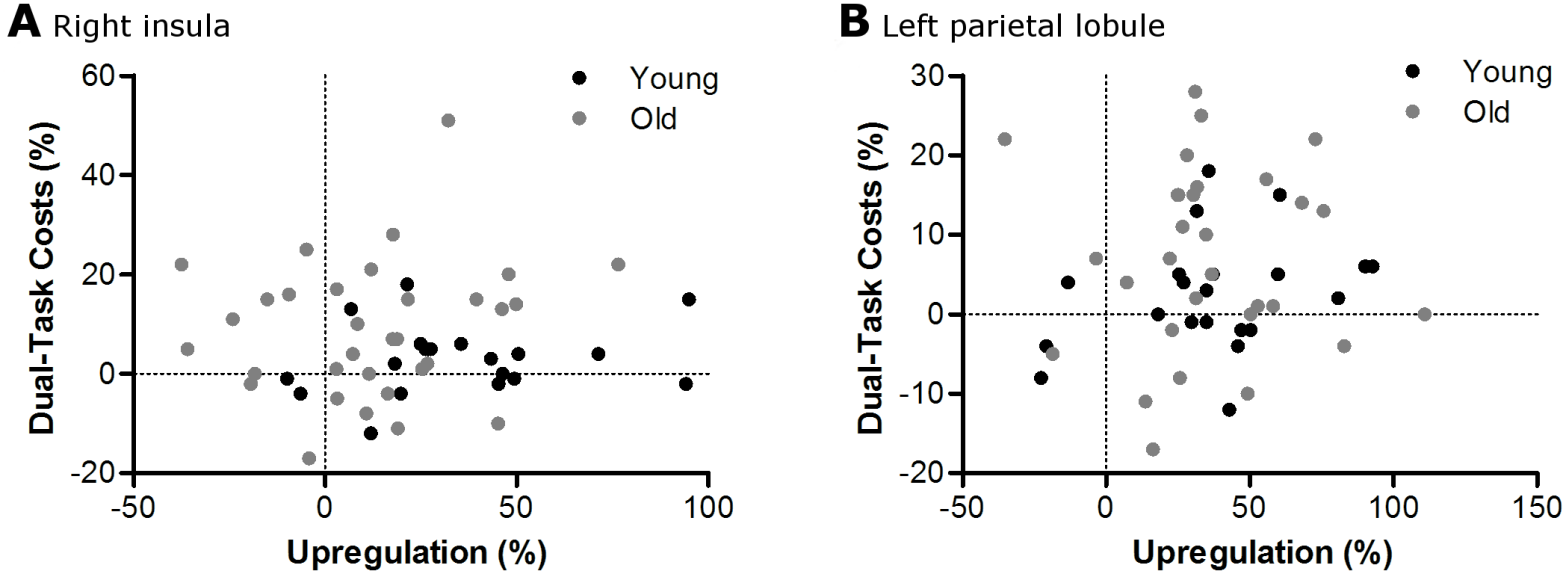
Scatter plots showing no relationship between the upregulation in BOLD response and the performance decline from single- to dual-tasking in the right insula (A) and the left parietal lobule (B).

In the right insula, there was less upregulation from single- to dual-task in old compared with young adults (t(52) = 3.2, p = 0.009). In the left parietal lobule, upregulation was not different between age groups (t(51) = 0.4, p = 1.000). Also mean upregulation across all ROI’s (cf Table 4) was not different between young and old adults (t(52) = 9.7, p = 0.136). There were no correlations between upregulation in the right insula, left parietal lobule, or mean upregulation across ROI’s, and DTC in young or old adults (Table 5 and Fig 5).

**Table 5:** Pearson correlation coefficients between upregulation in the right insula, left superior parietal lobule, and mean upregulation across all ROI’s, and balance, calculation, and mean dual-task costs (DTC). There were no significant correlations.

AAL location	Young						Old					
	Balance DTC		Calculation DTC		Mean DTC		Balance DTC		Calculation DTC		Mean DTC	
	Corr	P-value	Corr	P-value	Corr	P-value	Corr	P-value	Corr	P-value	Corr	P-value
R insula	0.136	0.567	0.138	0.563	0.189	0.424	0.260	0.151	-0.017	0.151	0.161	0.379
L superior parietal lobule	0.081	0.728	0.303	0.182	0.301	0.185	0.165	0.383	-0.150	0.429	-0.010	0.958
All ROI	0.337	0.135	0.156	0.498	0.299	0.187	0.108	0.562	0.038	0.839	0.128	0.492

## Discussion

We aimed to determine whether increased structural interference and/or dual-task specific activation underlies age-related dual-task decrements in the context of a balance task. As expected, we found greater dual-task cost (DTC), increased brain activation, and decreased upregulation (in the insula) in old compared with young adults. However, measures of brain activation and behavior did not correlate. We found no dual-task specific activation in either group. Therefore, under the current experimental conditions, neither structural interference nor dual-task specific activation could explain the age-related increase in DTC.

### Behavioral data

We consider our dual-task paradigm, comprising calculation plus simulated balancing, challenging and different in nature compared with previous studies that used finger sequencing [29] or visuomanual drawing and simple mental arithmetic [24]. Indeed, under such less challenging conditions it can be difficult to produce behavioral DTC [24]. We found lower performance levels and higher DTC in old compared with young adults when considering both calculation and balancing performance, but there was no significant effect of age on DTC when the tasks were considered separately. This may be because of a cancellation effect due to some subjects performing poorly on one task while other subjects performing poorly on the other task. Indeed, when looking at the individual scores, low performers on one task were not necessarily low performers on the other task. This means that in order to see the age effect on dual-task performance, performance changes in both tasks should be taken into account.

### Neural correlates of single-tasks

Although the condition vs. baseline contrasts give a general indication of task-related activity, such contrasts should be treated with caution, as spontaneous cortical activity may be present during the baseline condition. Nevertheless, activity patterns for the balance and calculation tasks were comparable to those reported in the literature. Imaging studies have often used arithmetic tasks and, as in the present study, consistently observed an increase in activation (as compared to baseline) of the frontal-parietal network [49]–[51]. Previous studies investigating the neural correlates of balance have used fMRI during mental imagery [39], [42], action observation [42], or a plantar flexion force control task [40], or PET-scans during actual standing [41]. Commonly activated areas consistent with the present study were the premotor cortex, prefrontal cortex, inferior and middle frontal gyrus, SMA, cerebellum, basal ganglia, thalamus, visual cortex, insula, and inferior parietal areas. The brain activation pattern we observed during the simulated balance task was remarkably similar to the activation pattern during the imagery and action observation of balancing [42], suggesting that perhaps our participants imagined themselves being the balancing avatar. The external validity of the simulated balance task was further supported by a significant correlation with center of pressure fluctuations during upright standing.

### Increased brain activation in old adults

Consistent with previous fMRI studies [43], [62]–[69], old adults exhibited greater brain activation than young adults in all tasks. Regarding balance tasks, age correlated with the activation of several multisensory areas during imaginary standing compared with lying [43]. We found age-related activation increases in both motor (precentral and paracentral gyri) and sensory (cuneus and precuneus) areas during the balance condition, possibly because our balance simulation was a real motor task instead of an imaginary motor task. During the calculation condition, the greatest cluster with an age-related increase in activation was located in the medial frontal gyrus. This area belongs to the prefrontal gyrus, in which age-related increases in activation during performance of cognitive tasks are well-known [62], [70].

The functional meaning of the increased brain activation with aging is still under debate [23]. One theory is that old adults use the additional activation to compensate for structural and functional decline, predicting a positive correlation between activation and behavioral performance. Alternatively, the dedifferentiation theory suggests that the more widespread activation pattern results from an age-related loss in the ability to selectively recruit brain areas. Consistent positive correlations between higher prefrontal activity and cognitive performance [71]–[74], as well as higher brain activity in various motor, parietal, frontal and cerebellar areas and motor performance [65], [75], strongly support the compensation theory. However, less neural specificity in visual areas for different stimulus categories (faces, houses, pseudowords, and chairs) in old compared with young adults [76], and negative correlations between age-related activation increases in ipsilateral motor areas and motor performance [64], [66], [77], show that at least in certain areas dedifferentiation occurs. As before [24], we found no correlation between activation in the areas over-activated by old adults and single-task performance, implying that the additional activation did not impair nor did it improve performance. This suggests an age-related spreading of brain activity beyond functional focus, favoring the dedifferentiation over the compensation theory. However, we must be cautious with such a conclusion because our analyses might have been underpowered.

### Dual-task specific activation

The literature is inconsistent regarding dual-task specific activation: whereas some studies did find evidence for dual-tasking requiring activation additional to the sum of the single-tasks [26]–[29], [78]–[80], others did not [17], [24], [30], [31]. Probably, the dual-task specific activation is dependent on the single-tasks, and there is no common executive control system that is active for all types of dual-tasking [21], [81]. In the current study, we did not find any dual-task specific activation in young or old adults. Therefore, it is unlikely that the age-related dual-task performance deficits were due to inadequate dual-task specific activation.

Although no additional activation was found during dual-tasking, we cannot exclude that the underlying processes, such as temporal dynamics, interactions between neuronal populations, or connectivity patterns, were different between single- and dual-tasking or between age groups. The assessment of such processes would require different neurophysiological measures, such as EEG or time-resolved fMRI.

### Structural interference

Regardless of the functional meaning, we hypothesized that the higher activation in old adults would cause structural interference and therefore greater DTC. However, we found no correlation between brain activation and DTC in the areas with age-related differences in activation. There are at least three possible explanations for this lack of correlation. First, the increased activation in old adults did not cause increased structural interference because the areas with higher activation were involved in only one of the two tasks. Second, old adults could still increase their brain activation from single- to dual-task, despite increased activation during single-tasks. Third, DTC was not related to structural interference.

To examine whether old adults exhibited greater structural interference, we performed a series of ROI analyses. Eight brain regions were selected because of their involvement in both tasks in both age groups. All regions, except the cerebellum, were expected to be activated in both tasks based on literature. We found an age by condition interaction in the right insula and left superior parietal lobule. Only in the right insula, however, there was an age-related increase in structural interference, quantified as a reduced activation-upregulation from single- to dual-task. However, the amount of upregulation was not correlated with DTC, suggesting that dual-task performance was not affected by the structural interference. It may be that the age-related increase in structural interference in the right insula was too small to cause DTC, or that insular activation was not essential for task performance. Although that would explain the lack of correlation between upregulation and DTC, the observed increase in DTC with aging remains unexplained. Therefore it seems that, at least under the current experimental conditions, age-related increases in DTC were not due to greater structural interference.

### Alternative theories

As neither structural interference nor dual-task specific activation could explain the age-related deficit in dual-task performance, we must consider alternative theories that could not be tested with the current experimental setup. One theory explaining DTC is the cross-talk model [82]. Cross-talk occurs when the effects of processing one task interfere with processing the other, and is therefore dependent on the content-based overlap between tasks and stimulus-response modalities [83], [84]. For example, DTC increased when the non-target words in one visual search task belonged to the target category in the other visual search task [83]. We believe that cross-talk was not a critical element in our experiment, as the tasks used did not have any content-based overlap, or incompatible or shared stimulus-response modalities. Moreover, there is no reason to think that age would affect the amount of cross-talk.

Another theory for DTC is that there is a central bottleneck that can perform certain processes only sequentially, resulting in serial queuing and time delays [85]. There is quite convincing evidence from behavioral and time-resolved fMRI studies that a central bottleneck is indeed present when two discrete choice reaction time tasks are performed with a short interstimulus interval [82], [86], [87]. It is possible that also during continuous tasks certain processes cannot be performed in parallel, requiring subjects to rapidly switch between tasks. As aging is associated with reduced processing speed, time delays may be more pronounced and interfere with smooth performance. Although evidence is lacking for healthy old adults, in multiple sclerosis patients processing speed indeed correlated with DTC when performing a cognitive task during gait or standing [88], [89]. To determine whether a central bottleneck can account for the age-related decline in dual-task performance, future research should use high-temporal resolution measures, such as time-resolved fMRI.

### Sample size and methodological considerations

Low statistical power could underlie the absence of correlations between fMRI measures and DTC. However, the number of participants in our study (32 old adults, 23 young adults) is high compared with similar studies [5], [24], [90], [91]. Furthermore, the scatter plots in Fig 5 show no trend for the negative correlation that would be expected based on the structural interference hypothesis and even show a tendency towards a positive correlation. Another methodological aspect to consider is whether the selection of tasks was optimal to test our hypothesis. The fact that we, in contrary to a previous study [24], did find increased dual-task costs and decreased upregulation with aging shows that the tasks were challenging enough and did cause structural interference. We therefore believe that the negative findings in the present study were not due to methodological limitations.

Another factor that may have influenced our results is head movement in the scanner, which was similar between conditions (F(1.3,70) =, p = 0.214), and higher in old compared with young adults (F(1,53) = 28, p < 0.001). Therefore, it must remain speculative whether missing DT-specific activation in old adults might also be due to increased noise in the data caused by head movements. Moreover, in old adults, head movements correlated with DTC (r = 0.477, p = 0.006), but not with single task performance (balance: r = 0.079, p = 0.669; calculation: r = -0.140, p = 0.446), complicating the interpretation of such data. In future studies, better control of head movements is thus required. However, we do believe that this had only a minor effect on our results, as we controlled for head movement by realigning the scans and using the obtained motion parameters as covariates in the fMRI analysis. In addition, we excluded subjects with excessive or task-related head movement.

Lastly, age-related changes in neurovascular coupling and morphology could have affected some of our results. As such changes should affect all conditions equally [92], it is highly unlikely that our main interest, the interaction between age and conditions, was affected. Regarding the group comparisons, we found a general increase in brain activation with aging, whereas age-related changes in neurovascular coupling and morphology would cause a reduction in the BOLD response [93]. Therefore, we assume that such changes did not affect our conclusions, although the age effect may be underestimated.

### Conclusions

We hypothesized that the age-related increase in brain activation during single-tasks would result in a reduced residual capacity, causing increased structural interference when performing two tasks simultaneously. Although we found increased brain activation, reduced upregulation from single- to dual-task, and greater DTC in old compared with young adults, we did not find any correlations between the fMRI measures and DTC. There was no dual-task specific activation in either age group. Therefore, it seems unlikely that the greater DTC in old adults were due to increased structural interference or differences in dual-task specific activation. A promising future research topic is to determine whether processing bottlenecks are present during continuous dual-tasks using high-temporal resolution measures (e.g., time-resolved fMRI).

## Supporting information

S1 Appendix Deactivation. MNI coordinates and t-values of the local maxima with significant deactivation during balance, calculation and dual-tasking (p < 0.05; FWE corrected for multiple comparisons). Voxel size is 3x3x3 mm.
(DOCX)

S2 Appendix MoCa. Montreal cognitive asessment (max. score of 30).
(DOCX)

S3 Appendix Behavioral data. Root-mean-square error (RMSE) angle between the avatar and the vertical during balance and dual-tasking, percentage trials with a correct answer on the calculation task during calculation and dual-tasking, and performance decline from single- to dual-tasking.
(DOCX)

S4 Appendix Upregulation. (DOCX)

S5 Appendix Validation. Center of pressure (CoP) velocity during normal standing and root-mean-square error (RMSE) angle between the avatar and the vertical during simulated standing.
(DOCX)

## References

[1] Lajoie Y., Teasdale N., Bard C., and Fleury M., “Attentional demands for static and dynamic equilibrium.,” *Exp*. *brain Res*., vol. 97, no. 1, pp. 139–44, 1 1993. 8131825 10.1007/BF00228824

[2] Riley M. A., Baker A. A., Schmit J. M., and Weaver E., “Effects of visual and auditory short-term memory tasks on the spatiotemporal dynamics and variability of postural sway.,” *J*. *Mot*. *Behav*., vol. 37, no. 4, pp. 311–24, 7 2005. 15967756 10.3200/JMBR.37.4.311-324

[3] Siu K.-C. and Woollacott M. H., “Attentional demands of postural control: the ability to selectively allocate information-processing resources.,” *Gait Posture*, vol. 25, no. 1, pp. 121–6, 1 2007. 16554158 10.1016/j.gaitpost.2006.02.002

[4] Maki B. E. and McIlroy W. E., “Influence of arousal and attention on the control of postural sway.,” *J*. *Vestib*. *Res*., vol. 6, no. 1, pp. 53–9, 1 1996. 8719510

[5] Hartley A. A., “Age differences in dual-task interference are localized to response-generation processes.,” *Psychol*. *Aging*, vol. 16, no. 1, pp. 47–54, 3 2001. 11302367 10.1037/0882-7974.16.1.47

[6] Maquestiaux F., Hartley A. A., and Bertsch J., “Can practice overcome age-related differences in the psychological refractory period effect?,” *Psychol*. *Aging*, vol. 19, no. 4, pp. 649–67, 12 2004. 15584790 10.1037/0882-7974.19.4.649

[7] Maylor E. A. and Wing A. M., “Age differences in postural stability are increased by additional cognitive demands.,” *J*. *Gerontol*. *B*. *Psychol*. *Sci*. *Soc*. *Sci*., vol. 51, no. 3, pp. P143–54, 5 1996. 8620354 10.1093/geronb/51b.3.p143

[8] Ruffieux J., Keller M., Lauber B., and Taube W., “Changes in Standing and Walking Performance Under Dual-Task Conditions Across the Lifespan.,” *Sports Med*., vol. 45, no. 12, pp. 1739–58, 12 2015. 26253187 10.1007/s40279-015-0369-9 PMC4656695

[9] Teasdale N., Bard C., LaRue J., and Fleury M., “On the cognitive penetrability of posture control.,” *Exp*. *Aging Res*., vol. 19, no. 1, pp. 1–13, 1 1993. 8444263 10.1080/03610739308253919

[10] Voelcker-Rehage C., Stronge A. J., and Alberts J. L., “Age-related differences in working memory and force control under dual-task conditions.,” *Neuropsychol*. *Dev*. *Cogn*. *B*. *Aging*. *Neuropsychol*. *Cogn*., vol. 13, no. 3–4, pp. 366–84, 1 2006. 16887779 10.1080/138255890969339

[11] Gill D. P., Gregory M. A., Zou G., Liu-Ambrose T., Shigematsu R., Hachinski V., et al, “The Healthy Mind, Healthy Mobility Trial: A Novel Exercise Program for Older Adults.,” *Med*. *Sci*. *Sports Exerc*., vol. 48, no. 2, pp. 297–306, 2 2016. 26285025 10.1249/MSS.0000000000000758

[12] Granacher U., Muehlbauer T., Zahner L., Gollhofer A., and Kressig R. W., “Comparison of traditional and recent aproaches in the promotion of balance and strength in older adults,” *Sport*. *Med*., vol. 41, no. 5, pp. 377–400, 2011. 10.2165/11539920-000000000-00000 21510715

[13] Silsupadol P., Shumway-Cook A., Lugade V., van Donkelaar P., Chou L., Mayr U., et al, “Effects of single-task versus dual-task training on balance performance in older adults: a double-blind, randomized controlled trial.,” *Arch*. *Phys*. *Med*. *Rehabil*., vol. 90, no. 3, pp. 381–7, 3 2009. 19254600 10.1016/j.apmr.2008.09.559 PMC2768031

[14] Wollesen B., Voelcker-Rehage C., Willer J., Zech A., and Mattes K., “Feasibility study of dual-task-managing training to improve gait performance of older adults.,” *Aging Clin*. *Exp*. *Res*., vol. 27, no. 4, pp. 447–55, 8 2015. 25556156 10.1007/s40520-014-0301-4

[15] Just M. A., Carpenter P. A., Keller T. A., Emery L., Zajac H., and Thulborn K. R., “Interdependence of nonoverlapping cortical systems in dual cognitive tasks.,” *Neuroimage*, vol. 14, no. 2, pp. 417–26, 8 2001. 11467915 10.1006/nimg.2001.0826

[16] Just M. A., Keller T. A., and Cynkar J., “A decrease in brain activation associated with driving when listening to someone speak.,” *Brain Res*., vol. 1205, pp. 70–80, 4 2008. 18353285 10.1016/j.brainres.2007.12.075 PMC2713933

[17] Klingberg T. and Roland P. E., “Interference between two concurrent tasks is associated with activation of overlapping fields in the cortex.,” *Brain Res*. *Cogn*. *Brain Res*., vol. 6, no. 1, pp. 1–8, 7 1997. 9395845 10.1016/s0926-6410(97)00010-4

[18] Menant J. C., Sturnieks D. L., Brodie M. A. D., Smith S. T., and Lord S. R., “Visuospatial tasks affect locomotor control more than nonspatial tasks in older people.,” *PLoS One*, vol. 9, no. 10, p. e109802, 1 2014. 25285913 10.1371/journal.pone.0109802 PMC4186860

[19] Nijboer M., Taatgen N. A., Brands A., Borst J. P., and van Rijn H., “Decision making in concurrent multitasking: do people adapt to task interference?,” *PLoS One*, vol. 8, no. 11, p. e79583, 1 2013. 24244527 10.1371/journal.pone.0079583 PMC3823623

[20] Salvucci D. D. and Taatgen N. A., “Threaded cognition: an integrated theory of concurrent multitasking.,” *Psychol*. *Rev*., vol. 115, no. 1, pp. 101–30, 1 2008. 18211187 10.1037/0033-295X.115.1.101

[21] Nijboer M., Borst J., van Rijn H., and Taatgen N., “Single-task fMRI overlap predicts concurrent multitasking interference.,” *Neuroimage*, vol. 100, pp. 60–74, 10 2014. 24911376 10.1016/j.neuroimage.2014.05.082

[22] Papegaaij S., Taube W., Baudry S., Otten E., and Hortobágyi T., “Aging causes a reorganization of cortical and spinal control of posture,” *Frontiers in Aging Neuroscience*, vol. 6, no 3 Frontiers Media SA, 2014. 10.3389/fnagi.2014.00028 PMC3939445 24624082

[23] Seidler R. D. et al, “Motor control and aging: links to age-related brain structural, functional, and biochemical effects.,” *Neurosci*. *Biobehav*. *Rev*., vol. 34, no. 5, pp. 721–33, 4 2010. 19850077 10.1016/j.neubiorev.2009.10.005 PMC2838968

[24] Van Impe A., Coxon J. P., Goble D. J., Wenderoth N., and Swinnen S. P., “Age-related changes in brain activation underlying single- and dual-task performance: visuomanual drawing and mental arithmetic.,” *Neuropsychologia*, vol. 49, no. 9, pp. 2400–9, 7 2011. 21536055 10.1016/j.neuropsychologia.2011.04.016

[25] Voelcker-Rehage C. and Alberts J. L., “Effect of motor practice on dual-task performance in older adults.,” *J*. *Gerontol*. *B*. *Psychol*. *Sci*. *Soc*. *Sci*., vol. 62, no. 3, pp. P141–8, 5 2007. 17507581 10.1093/geronb/62.3.p141

[26] D’Esposito M., Detre J. A., Alsop D. C., Shin R. K., Atlas S., and Grossman M., “The neural basis of the central executive system of working memory.,” *Nature*, vol. 378, no. 6554, pp. 279–81, 11 1995. 7477346 10.1038/378279a0

[27] Dreher J.-C. and Grafman J., “Dissociating the roles of the rostral anterior cingulate and the lateral prefrontal cortices in performing two tasks simultaneously or successively.,” *Cereb*. *Cortex*, vol. 13, no. 4, pp. 329–39, 4 2003. 12631562 10.1093/cercor/13.4.329

[28] Szameitat A. J., Schubert T., Müller K., and Von Cramon D. Y., “Localization of executive functions in dual-task performance with fMRI.,” *J*. *Cogn*. *Neurosci*., vol. 14, no. 8, pp. 1184–99, 11 2002. 12495525 10.1162/089892902760807195

[29] Wu T., Liu J., Hallett M., Zheng Z., and Chan P., “Cerebellum and integration of neural networks in dual-task processing.,” *Neuroimage*, vol. 65, pp. 466–75, 1 2013. 23063842 10.1016/j.neuroimage.2012.10.004 PMC4173076

[30] Adcock R. A., Constable R. T., Gore J. C., and Goldman-Rakic P. S., “Functional neuroanatomy of executive processes involved in dual-task performance.,” *Proc*. *Natl*. *Acad*. *Sci*. *U*. *S*. *A*., vol. 97, no. 7, pp. 3567–72, 3 2000. 10725387 10.1073/pnas.060588897 PMC16280

[31] Johannsen L., Li K. Z. H., Chechlacz M., Bibi A., Kourtzi Z., and Wing A. M., “Functional neuroimaging of the interference between working memory and the control of periodic ankle movement timing.,” *Neuropsychologia*, vol. 51, no. 11, pp. 2142–53, 9 2013. 23876923 10.1016/j.neuropsychologia.2013.07.009 PMC4410789

[32] Pahor M., Guralnik J. M., Ambrosius W. T., Blair S., Bonds D. E., Church T. S., et al, “Effect of structured physical activity on prevention of major mobility disability in older adults: the LIFE study randomized clinical trial.,” *JAMA*, vol. 311, no. 23, pp. 2387–96, 6 2014. 24866862 10.1001/jama.2014.5616 PMC4266388

[33] Shinkai S., Watanabe S., Kumagai S., Fujiwara Y., Amano H., Yoshida H., et al, “Walking speed as a good predictor for the onset of functional dependence in a Japanese rural community population.,” *Age Ageing*, vol. 29, no. 5, pp. 441–6, 9 2000. 11108417 10.1093/ageing/29.5.441

[34] Talley K. M. C., Wyman J. F., Gross C. R., Lindquist R. A., and Gaugler J. E., “Change in Balance Confidence and Its Associations With Increasing Disability in Older Community-Dwelling Women at Risk for Falling.,” *J*. *Aging Health*, vol. 26, no. 4, pp. 616–636, 3 2014. 24667106 10.1177/0898264314526619 PMC5659850

[35] Beauchet O., Annweiler C., Dubost V., Allali G., Kressig R. W., Bridenbaugh S., et al, “Stops walking when talking: a predictor of falls in older adults?,” *Eur*. *J*. *Neurol*., vol. 16, no. 7, pp. 786–95, 7 2009. 19473368 10.1111/j.1468-1331.2009.02612.x

[36] Walshe E. A., Patterson M. R., Commins S., and Roche R. A. P., “Dual-task and electrophysiological markers of executive cognitive processing in older adult gait and fall-risk.,” *Front*. *Hum*. *Neurosci*., vol. 9, p. 200, 1 2015. 25941481 10.3389/fnhum.2015.00200 PMC4400911

[37] Beauchet O., Annweiler C., Allali G., Berrut G., Herrmann F. R., and Dubost V., “Recurrent falls and dual task-related decrease in walking speed: is there a relationship?,” *J*. *Am*. *Geriatr*. *Soc*., vol. 56, no. 7, pp. 1265–9, 7 2008. 18510582 10.1111/j.1532-5415.2008.01766.x

[38] Wang X., Pi Y., Chen P., Liu Y., Wang R., and Chan C., “Cognitive motor interference for preventing falls in older adults: a systematic review and meta-analysis of randomised controlled trials.,” *Age Ageing*, vol. 44, no. 2, pp. 205–12, 3 2015. 25377745 10.1093/ageing/afu175

[39] Jahn K., Deutschländer A., Stephan T., Strupp M., Wiesmann M., and Brandt T., “Brain activation patterns during imagined stance and locomotion in functional magnetic resonance imaging.,” *Neuroimage*, vol. 22, no. 4, pp. 1722–31, 8 2004. 15275928 10.1016/j.neuroimage.2004.05.017

[40] Karim H. T., Sparto P. J., Aizenstein H. J., Furman M. J., Huppert T. J., Erickson K. I., et al, “Functional MR imaging of a simulated balance task.,” *Brain Res*., vol. 1555, pp. 20–7, 3 2014. 24480476 10.1016/j.brainres.2014.01.033 PMC4001860

[41] Ouchi Y., Okada H., Yoshikawa E., Nobezawa S., and Futatsubashi M., “Brain activation during maintenance of standing postures in humans.,” *Brain*, vol. 122 (Pt 2, pp. 329–38, 2 1999. 10071060 10.1093/brain/122.2.329

[42] Taube W., Mouthon M., Leukel C., Hoogewoud H.-M., Annoni J.-M., and Keller M., “Brain activity during observation and motor imagery of different balance tasks: an fMRI study.,” *Cortex*., vol. 64, pp. 102–14, 3 2015. 25461711 10.1016/j.cortex.2014.09.022

[43] Zwergal A., Linn J., Xiong G., Brandt T., Strupp M., and Jahn K., “Aging of human supraspinal locomotor and postural control in fMRI.,” *Neurobiol*. *Aging*, vol. 33, no. 6, pp. 1073–84, 6 2012. 21051105 10.1016/j.neurobiolaging.2010.09.022

[44] Kouzaki M. and Shinohara M., “Steadiness in plantar flexor muscles and its relation to postural sway in young and elderly adults.,” *Muscle Nerve*, vol. 42, no. 1, pp. 78–87, 7 2010. 20544908 10.1002/mus.21599 PMC4590785

[45] Mello E. M., Magalhães F. H., and Kohn A. F., “Larger plantar flexion torque variability implies less stable balance in the young: an association affected by knee position.,” *Hum*. *Mov*. *Sci*., vol. 32, no. 6, pp. 1310–24, 12 2013. 24060221 10.1016/j.humov.2013.05.004

[46] Boisgontier M. P., Beets I. A. M., Duysens J., Nieuwboer A., Krampe R. T., and Swinnen S. P., “Age-related differences in attentional cost associated with postural dual tasks: increased recruitment of generic cognitive resources in older adults.,” *Neurosci*. *Biobehav*. *Rev*., vol. 37, no. 8, pp. 1824–37, 9 2013. 23911924 10.1016/j.neubiorev.2013.07.014

[47] Godde B. and Voelcker-Rehage C., “More automation and less cognitive control of imagined walking movements in high- versus low-fit older adults.,” *Front*. *Aging Neurosci*., vol. 2, 1 2010. 10.3389/fnagi.2010.00139 PMC2944669 20877433

[48] Cowell S. F., Egan G. F., Code C., Harasty J., and Watson J. D., “The functional neuroanatomy of simple calculation and number repetition: A parametric PET activation study.,” *Neuroimage*, vol. 12, no. 5, pp. 565–73, 11 2000. 11034863 10.1006/nimg.2000.0640

[49] Kong J., Wang C., Kwong K., Vangel M., Chua E., and Gollub R., “The neural substrate of arithmetic operations and procedure complexity.,” *Brain Res*. *Cogn*. *Brain Res*., vol. 22, no. 3, pp. 397–405, 3 2005. 15722210 10.1016/j.cogbrainres.2004.09.011

[50] Vansteensel M. J., Bleichner M. G., Freudenburg Z. V., Hermes D., Aarnoutse E. J., Leijten F. S. S., et al, “Spatiotemporal characteristics of electrocortical brain activity during mental calculation.,” *Hum*. *Brain Mapp*., vol. 35, no. 12, pp. 5903–20, 12 2014. 25044370 10.1002/hbm.22593 PMC6426240

[51] Yi-Rong N., Si-Yun S., Zhou-Yi G., Si-Run L., Yun B., Song-Hao L., et al, “Dissociated brain organization for two-digit addition and subtraction: an fMRI investigation.,” *Brain Res*. *Bull*., vol. 86, no. 5–6, pp. 395–402, 11 2011. 21906662 10.1016/j.brainresbull.2011.08.016

[52] Niemann C., Godde B., and Voelcker-Rehage C., “Senior Dance Experience, Cognitive Performance, and Brain Volume in Older Women.,” *Neural Plast*., vol. 2016, p. 9837321, 1 2016. 27738528 10.1155/2016/9837321 PMC5055974

[53] Kenny R. A., Coen R. F., Frewen J., Donoghue O. A., Cronin H., and Savva G. M., “Normative values of cognitive and physical function in older adults: findings from the Irish Longitudinal Study on Ageing.,” *J*. *Am*. *Geriatr*. *Soc*., vol. 61 Suppl 2, pp. S279–90, 5 2013. 23662720 10.1111/jgs.12195

[54] Damian A. M., Jacobson S. A., Hentz J. G., Belden C. M., Shill H. A., Sabbagh M. N., et al, “The Montreal Cognitive Assessment and the mini-mental state examination as screening instruments for cognitive impairment: item analyses and threshold scores.,” *Dement*. *Geriatr*. *Cogn*. *Disord*., vol. 31, no. 2, pp. 126–31, 1 2011. 21282950 10.1159/000323867

[55] O’Caoimh R., Timmons S., and Molloy D. W., “Screening for Mild Cognitive Impairment: Comparison of ‘MCI Specific’ Screening Instruments.,” *J*. *Alzheimers*. *Dis*., vol. 51, no. 2, pp. 619–29, 1 2016. 26890758 10.3233/JAD-150881 PMC4927818

[56] Waldron‐Perrine B. and Axelrod B., “Determining an appropriate cutting score for indication of impairment on the Montreal Cognitive Assessment,” *Int*. *J*., 2012. 10.1002/gps.3768 22228412

[57] Amaro E. and Barker G. J., “Study design in fMRI: Basic principles,” *Brain* Cogn., vol. 60, no. 3, pp. 220–232, 2006. 16427175 10.1016/j.bandc.2005.11.009

[58] Bock O., “Dual-task costs while walking increase in old age for some, but not for other tasks: an experimental study of healthy young and elderly persons.,” *J*. *Neuroeng*. *Rehabil*., vol. 5, p. 27, 11 2008. 19014544 10.1186/1743-0003-5-27 PMC2596160

[59] J. Tukey, “Exploratory data analysis,” 1977.

[60] Hoaglin D. C. and Iglewicz B., “Fine-Tuning Some Resistant Rules for Outlier Labeling,” *J*. *Am*. *Stat*. *Assoc*., 3 1987.

[61] Szameitat A. J., Schubert T., and Müller H. J., “How to test for dual-task-specific effects in brain imaging studies—an evaluation of potential analysis methods.,” *Neuroimage*, vol. 54, no. 3, pp. 1765–73, 2 2011. 20688175 10.1016/j.neuroimage.2010.07.069

[62] Cabeza R., “Cognitive neuroscience of aging: contributions of functional neuroimaging.,” *Scand*. *J*. *Psychol*., vol. 42, no. 3, pp. 277–86, 7 2001. 11501741 10.1111/1467-9450.00237

[63] Hamacher D., Herold F., Wiegel P., Hamacher D., and Schega L., “Brain activity during walking: A systematic review.,” *Neurosci*. *Biobehav*. *Rev*., vol. 57, pp. 310–27, 10 2015. 26306029 10.1016/j.neubiorev.2015.08.002

[64] Langan J., Peltier S. J., Bo J., Fling B. W., Welsh R. C., and Seidler R. D., “Functional implications of age differences in motor system connectivity.,” *Front*. *Syst*. *Neurosci*., vol. 4, p. 17, 1 2010. 20589101 10.3389/fnsys.2010.00017 PMC2893009

[65] Mattay V. S., Fera F., Tessitore A., Hariri A. R., Das S., Callicott J. H., et al, “Neurophysiological correlates of age-related changes in human motor function.,” *Neurology*, vol. 58, no. 4, pp. 630–5, 2 2002. 11865144 10.1212/wnl.58.4.630

[66] McGregor K. M., Zlatar Z., Klein E., Sudhyadhom A., Bauer A., Phan S., et al, “Physical activity and neural correlates of aging: a combined TMS/fMRI study.,” *Behav*. *Brain Res*., vol. 222, no. 1, pp. 158–68, 9 2011. 21440574 10.1016/j.bbr.2011.03.042 PMC3713467

[67] Park D. C. and McDonough I. M., “The Dynamic Aging Mind: Revelations From Functional Neuroimaging Research.,” *Perspect*. *Psychol*. *Sci*., vol. 8, no. 1, pp. 62–7, 1 2013. 26172252 10.1177/1745691612469034

[68] Reuter-Lorenz P. A., Jonides J., Smith E. E., Hartley A., Miller A., Marshuetz C., et al, “Age differences in the frontal lateralization of verbal and spatial working memory revealed by PET.,” *J*. *Cogn*. *Neurosci*., vol. 12, no. 1, pp. 174–87, 1 2000. 10769314 10.1162/089892900561814

[69] Ward N. S. and Frackowiak R. S. J., “Age-related changes in the neural correlates of motor performance.,” *Brain*, vol. 126, no. Pt 4, pp. 873–88, 4 2003. 12615645 10.1093/brain/awg071 PMC3717766

[70] Davis S. W., Dennis N. A., Daselaar S. M., Fleck M. S., and Cabeza R., “Que PASA? The posterior-anterior shift in aging.,” *Cereb*. *Cortex*, vol. 18, no. 5, pp. 1201–9, 5 2008. 17925295 10.1093/cercor/bhm155 PMC2760260

[71] Cabeza R., Anderson N. D., Locantore J. K., and McIntosh A. R., “Aging gracefully: compensatory brain activity in high-performing older adults.,” *Neuroimage*, vol. 17, no. 3, pp. 1394–402, 11 2002. 12414279 10.1006/nimg.2002.1280

[72] Cherry B. and Adamson M., “Aging and individual variation in interhemispheric collaboration and hemispheric asymmetry,” *Aging*, *Neuropsychol*. *…*, 2005. 10.1080/17444128.2005.10367004 28486832

[73] Fera F., Weickert T. W., Goldberg T. E., Tessitore A., Hariri A., Das S., et al, “Neural mechanisms underlying probabilistic category learning in normal aging.,” *J*. *Neurosci*., vol. 25, no. 49, pp. 11340–8, 12 2005. 16339029 10.1523/JNEUROSCI.2736-05.2005 PMC6725912

[74] Reuter-Lorenz P. A., Stanczak L., and Miller A. C., “Neural Recruitment and Cognitive Aging: Two Hemispheres Are Better Than One, Especially as You Age,” *Psychol*. *Sci*., vol. 10, no. 6, pp. 494–500, 11 1999.

[75] Heuninckx S., Wenderoth N., and Swinnen S. P., “Systems neuroplasticity in the aging brain: recruiting additional neural resources for successful motor performance in elderly persons.,” *J*. *Neurosci*., vol. 28, no. 1, pp. 91–9, 1 2008. 18171926 10.1523/JNEUROSCI.3300-07.2008 PMC6671150

[76] Park D. C., Polk T. A., Park R., Minear M., Savage A., and Smith M. R., “Aging reduces neural specialization in ventral visual cortex.,” *Proc*. *Natl*. *Acad*. *Sci*. *U*. *S*. *A*., vol. 101, no. 35, pp. 13091–5, 8 2004. 15322270 10.1073/pnas.0405148101 PMC516469

[77] Riecker A., Gröschel K., Ackermann H., Steinbrink C., Witte O., and Kastrup A., “Functional significance of age-related differences in motor activation patterns.,” *Neuroimage*, vol. 32, no. 3, pp. 1345–54, 9 2006. 16798017 10.1016/j.neuroimage.2006.05.021

[78] Herath P., Klingberg T., Young J., Amunts K., and Roland P., “Neural correlates of dual task interference can be dissociated from those of divided attention: an fMRI study.,” *Cereb*. *Cortex*, vol. 11, no. 9, pp. 796–805, 9 2001. 11532885 10.1093/cercor/11.9.796

[79] Schubert T. and Szameitat A. J., “Functional neuroanatomy of interference in overlapping dual tasks: an fMRI study.,” *Brain Res*. *Cogn*. *Brain Res*., vol. 17, no. 3, pp. 733–46, 10 2003. 14561459 10.1016/s0926-6410(03)00198-8

[80] Wu T. and Hallett M., “Neural correlates of dual task performance in patients with Parkinson’s disease.,” *J*. *Neurol*. *Neurosurg*. *Psychiatry*, vol. 79, no. 7, pp. 760–6, 7 2008. 18006652 10.1136/jnnp.2007.126599

[81] Salo E., Rinne T., Salonen O., and Alho K., “Brain activations during bimodal dual tasks depend on the nature and combination of component tasks.,” *Front*. *Hum*. *Neurosci*., vol. 9, p. 102, 1 2015. 25767443 10.3389/fnhum.2015.00102 PMC4341542

[82] Pashler H., “Dual-task interference in simple tasks: data and theory.,” *Psychol*. *Bull*., vol. 116, no. 2, pp. 220–44, 9 1994. 7972591 10.1037/0033-2909.116.2.220

[83] Navon D. and Miller J., “Role of outcome conflict in dual-task interference.,” *J*. *Exp*. *Psychol*. *Hum*. *Percept*. *Perform*., vol. 13, no. 3, pp. 435–48, 8 1987. 2958592 10.1037//0096-1523.13.3.435

[84] Stelzel C. and Schubert T., “Interference effects of stimulus-response modality pairings in dual tasks and their robustness.,” *Psychol*. *Res*., vol. 75, no. 6, pp. 476–90, 11 2011. 21811837 10.1007/s00426-011-0368-x

[85] Pashler H., “Processing stages in overlapping tasks: evidence for a central bottleneck.,” *J*. *Exp*. *Psychol*. *Hum*. *Percept*. *Perform*., vol. 10, no. 3, pp. 358–77, 6 1984. 6242412 10.1037//0096-1523.10.3.358

[86] Dux P. E., Ivanoff J., Asplund C. L., and Marois R., “Isolation of a central bottleneck of information processing with time-resolved FMRI.,” *Neuron*, vol. 52, no. 6, pp. 1109–20, 12 2006. 17178412 10.1016/j.neuron.2006.11.009 PMC2527865

[87] Sigman M. and Dehaene S., “Parsing a cognitive task: a characterization of the mind’s bottleneck.,” *PLoS Biol*., vol. 3, no. 2, p. e37, 2 2005. 15719056 10.1371/journal.pbio.0030037 PMC546328

[88] Prosperini L., Castelli L., Sellitto G., De Luca F., De Giglio L., Gurreri F., et al, “Investigating the phenomenon of ‘cognitive-motor interference’ in multiple sclerosis by means of dual-task posturography.,” *Gait Posture*, vol. 41, no. 3, pp. 780–5, 3 2015. 25770078 10.1016/j.gaitpost.2015.02.002

[89] Sosnoff J. J., Socie M. J., Sandroff B. M., Balantrapu S., Suh Y., Pula J. H., et al, “Mobility and cognitive correlates of dual task cost of walking in persons with multiple sclerosis.,” *Disabil*. *Rehabil*., vol. 36, no. 3, pp. 205–9, 1 2014. 23597000 10.3109/09638288.2013.782361

[90] Goble D. J., Coxon J. P., Van Impe A., Geurts M., Doumas M., Wenderoth N. et al, “Brain activity during ankle proprioceptive stimulation predicts balance performance in young and older adults.,” *J*. *Neurosci*., vol. 31, no. 45, pp. 16344–52, 11 2011. 22072686 10.1523/JNEUROSCI.4159-11.2011 PMC6633212

[91] Van Impe A., Bruijn S., Coxon J. P., Wenderoth N., Sunaert S., Duysens J., et al, “Age-related neural correlates of cognitive task performance under increased postural load.,” *Age (Dordr)*., vol. 35, no. 6, pp. 2111–24, 12 2013. 23274853 10.1007/s11357-012-9499-2 PMC3824995

[92] Samanez-Larkin G. R. and D’Esposito M., “Group comparisons: imaging the aging brain.,” *Soc*. *Cogn*. *Affect*. *Neurosci*., vol. 3, no. 3, pp. 290–7, 9 2008. 18846241 10.1093/scan/nsn029 PMC2563421

[93] D’Esposito M., Deouell L. Y., and Gazzaley A., “Alterations in the BOLD fMRI signal with ageing and disease: a challenge for neuroimaging,” *Nat*. *Rev*. *Neurosci*., vol. 4, no. 11, pp. 863–872, 11 2003. 14595398 10.1038/nrn1246

