# Supplementary material for: Neural correlates of motor-cognitive dual-tasking in young and old adults
Source: PLoS One. 2017 Dec 8;12(12):e0189025. doi: 10.1371/journal.pone.0189025 (PMC5722310; doi:10.1371/journal.pone.0189025)
Supplement: S1 Appendix — MNI coordinates and t-values of the local maxima with significant deactivation during balance, calculation and dual-tasking (p < 0.05; FWE corrected for multiple comparisons). Voxel size is 3x3x3 mm. (DOCX) [file pone.0189025.s001.docx]

| AAL location | Side | Dual-task | | | |  | Balance | | | |  | Calculation | | | |
| --- | --- | --- | --- | --- | --- | --- | --- | --- | --- | --- | --- | --- | --- | --- | --- |
|  |  | MNI coordinates | | | t-value |  | MNI coordinates | | | t-value |  | MNI coordinates | | | t-value |
|  |  | x | y | z |  |  | x | y | z |  |  | x | y | z |  |
| Medial frontal gyrus | L | 0 | 60 | 15 | 12.3 |  |  |  |  |  |  | -3 | 60 | 15 | 17.1 |
|  | R | 12 | 33 | 60 | 7.7 |  |  |  |  |  |  | 12 | 42 | 51 | 10.4 |
| Superior frontal gyrus | L | -9 | 27 | 63 | 9.7 |  | -18 | 57 | 3 | 4.8 |  | -9 | 27 | 63 | 12.8 |
|  | R | 15 | 60 | 24 | 5.6 |  |  |  |  |  |  |  |  |  |  |
| Middle frontal gyrus | L | -39 | 18 | 51 | 5.2 |  |  |  |  |  |  | -36 | 18 | 48 | 7.9 |
| Inferior frontal gyrus (orbital part) | L |  |  |  |  |  |  |  |  |  |  | -36 | 33 | -12 | 5.8 |
|  | R |  |  |  |  |  |  |  |  |  |  | 36 | 18 | -21 | 5.5 |
| Inferior frontal gyrus (pars triangularis) | L | -54 | 30 | 9 | 4.9 |  |  |  |  |  |  | -54 | 30 | 9 | 7.1 |
| Medial orbitofrontal cortex | L | -3 | 51 | -6 | 10.7 |  |  |  |  |  |  | -3 | 51 | -6 | 14.0 |
| Insula | L | -27 | 15 | -18 | 6.5 |  |  |  |  |  |  | -27 | 15 | -18 | 6.3 |
|  | R | 39 | -12 | 18 | 5.6 |  |  |  |  |  |  | 42 | -9 | 3 | 5.6 |
| Anterior cingulate gyrus | L | 0 | 36 | 3 | 10.7 |  | 0 | 33 | 6 | 7.1 |  | 0 | 36 | 0 | 12.9 |
| Midcingulate area | L | 0 | -48 | 33 | 12.1 |  |  |  |  |  |  | 0 | -48 | 33 | 15.8 |
| Postcentral gyrus | L |  |  |  |  |  |  |  |  |  |  | -21 | -42 | 75 | 5.3 |
|  | R |  |  |  |  |  |  |  |  |  |  | 21 | -42 | 69 | 8.7 |
| Precuneus | L | -9 | -54 | 9 | 5.8 |  |  |  |  |  |  |  |  |  |  |
|  | R |  |  |  |  |  | 9 | -48 | 39 | 5.1 |  |  |  |  |  |
| Angular gyrus | L | -51 | -66 | 36 | 11.0 |  | -42 | -75 | 39 | 5.8 |  | -54 | -66 | 30 | 14.4 |
|  | R | 51 | -66 | 36 | 10.9 |  | 48 | -69 | 36 | 5.1 |  | 51 | -60 | 24 | 14.0 |
| Supramarginal gyrus | L |  |  |  |  |  |  |  |  |  |  | -63 | -30 | 33 | 5.6 |
|  | R |  |  |  |  |  |  |  |  |  |  | 60 | -24 | 30 | 7.1 |
| Superior temporal pole | L |  |  |  |  |  |  |  |  |  |  | -39 | 21 | -18 | 6.4 |
|  | R | 27 | 6 | -24 | 5.6 |  |  |  |  |  |  | 30 | 9 | -24 | 6.5 |
| Middle temporal pole | R | 48 | 18 | -33 | 5.0 |  |  |  |  |  |  | 48 | 18 | -33 | 5.7 |
| Middle temporal gyrus | L | -63 | -9 | -15 | 8.6 |  |  |  |  |  |  | -63 | -9 | -15 | 11.2 |
|  | R | 66 | -15 | -12 | 5.8 |  |  |  |  |  |  | 60 | -9 | -18 | 8.0 |
| Hippocampus | L | -27 | -21 | -18 | 5.8 |  |  |  |  |  |  | -27 | -18 | -18 | 6.7 |
| Parahippocampal gyrus | L | -27 | -42 | -9 | 5.7 |  |  |  |  |  |  |  |  |  |  |
|  | R | 24 | -36 | -12 | 5.4 |  |  |  |  |  |  | 21 | -36 | -12 | 6.0 |
| Amygdala | L | -18 | -3 | -18 | 5.3 |  |  |  |  |  |  | -18 | 0 | -18 | 7.1 |
|  | R |  |  |  |  |  |  |  |  |  |  | 24 | -3 | -18 | 6.4 |
| Olfactory cortex | L |  |  |  |  |  |  |  |  |  |  | 0 | 12 | -6 | 10.2 |
| Superior occipital gyrus | L |  |  |  |  |  |  |  |  |  |  | -15 | -96 | 27 | 6.3 |
| Middle occipital gyrus | L |  |  |  |  |  |  |  |  |  |  | -36 | -90 | 21 | 5.7 |
|  | R |  |  |  |  |  |  |  |  |  |  | 39 | -84 | 27 | 7.6 |
| Calcarine sulcus | L |  |  |  |  |  | -12 | -57 | 6 | 5.2 |  |  |  |  |  |
|  | R |  |  |  |  |  | 9 | -78 | 3 | 6.0 |  |  |  |  |  |
| Lingual gyrus | L |  |  |  |  |  | -9 | -78 | -3 | 5.5 |  | -24 | -45 | -9 | 6.5 |
| Cuneus | L | -6 | -93 | 30 | 6.1 |  | 0 | -87 | 33 | 10.0 |  | -9 | -90 | 36 | 4.9 |
|  | R | 6 | -87 | 33 | 5.8 |  |  |  |  |  |  | 21 | -87 | 39 | 4.9 |
| Caudate nucleus | L | -3 | 15 | -6 | 7.7 |  |  |  |  |  |  |  |  |  |  |
| Cerebellum (lobule 9) | R |  |  |  |  |  |  |  |  |  |  | 6 | -51 | -45 | 4.8 |
| Cerebellar hemisphere (crus I) | L |  |  |  |  |  |  |  |  |  |  | -27 | -81 | -33 | 8.0 |
|  | R | 27 | -81 | -33 | 7.8 |  |  |  |  |  |  | 24 | -84 | -33 | 8.8 |
| Cerebellar hemisphere (crus II) | L | -30 | -81 | -36 | 6.4 |  |  |  |  |  |  |  |  |  |  |

Supplementary material. MNI coordinates and t-values of the local maxima with significant deactivation during balance, calculation and dual-tasking (p < 0.05; FWE corrected for multiple comparisons). Voxel size is 3x3x3 mm.
